# Predator selection on multicomponent warning signals in an aposematic moth

**DOI:** 10.1093/beheco/arad097

**Published:** 2023-11-16

**Authors:** Liisa Hämäläinen, Georgina E Binns, Nathan S Hart, Johanna Mappes, Paul G McDonald, Louis G O’Neill, Hannah M Rowland, Kate D L Umbers, Marie E Herberstein

**Affiliations:** School of Natural Sciences, Macquarie University, 14 Eastern Road, North Ryde, NSW 2109, Australia; School of Science, Western Sydney University, Locked Bag 1797, Penrith, NSW 2751, Australia; Department of Biological and Environmental Science, University of Jyväskylä, PO Box 35, 40014 Jyväskylä, Finland; School of Natural Sciences, Macquarie University, 14 Eastern Road, North Ryde, NSW 2109, Australia; School of Natural Sciences, Macquarie University, 14 Eastern Road, North Ryde, NSW 2109, Australia; Organismal and Evolutionary Biology Research Programme, Faculty of Biological and Environmental Sciences, University of Helsinki, Viikinkaari 1, PO Box 65, 00014 Helsinki, Finland; School of Environmental and Rural Science, University of New England, Elm Avenue, Armidale, NSW 2351, Australia; School of Natural Sciences, Macquarie University, 14 Eastern Road, North Ryde, NSW 2109, Australia; Max Planck Institute for Chemical Ecology, Hans Knöll Straße 8, 07745 Jena, Germany; School of Science, Western Sydney University, Locked Bag 1797, Penrith, NSW 2751, Australia; Hawkesbury Institute for the Environment, Western Sydney University, Locked Bag 1797, Penrith, NSW 2751, Australia; School of Natural Sciences, Macquarie University, 14 Eastern Road, North Ryde, NSW 2109, Australia

**Keywords:** aposematism, color pattern, Lepidoptera, noisy miner, salience, warning signals

## Abstract

Aposematic prey advertise their unprofitability with conspicuous warning signals that are often composed of multiple color patterns. Many species show intraspecific variation in these patterns even though selection is expected to favor invariable warning signals that enhance predator learning. However, if predators acquire avoidance to specific signal components, this might relax selection on other aposematic traits and explain variability. Here, we investigated this idea in the aposematic moth *Amata nigriceps* that has conspicuous black and orange coloration. The size of the orange spots in the wings is highly variable between individuals, whereas the number and width of orange abdominal stripes remains consistent. We produced artificial moths that varied in the proportion of orange in the wings or the presence of abdominal stripes. We presented these to a natural avian predator, the noisy miner (*Manorina melanocephala)*, and recorded how different warning signal components influenced their attack decisions. When moth models had orange stripes on the abdomen, birds did not discriminate between different wing signals. However, when the stripes on the abdomen were removed, birds chose the model with smaller wing spots. In addition, we found that birds were more likely to attack moths with a smaller number of abdominal stripes. Together, our results suggest that bird predators primarily pay attention to the abdominal stripes of *A. nigriceps,* and this could relax selection on wing coloration. Our study highlights the importance of considering individual warning signal components if we are to understand how predation shapes selection on prey warning coloration.

## Teaser

Within-species variation in prey warning signals can be explained by some signal components being less important for predator foraging decisions. We investigated how orange wing spots and abdominal stripes of the aposematic moth, *Amata nigriceps* , influence predator attack decisions and found that generalist bird predators did not pay attention to wing spots when stripes were present. This could relax selection on consistent wing patterns and explain the observed variation in *A. nigriceps* wing coloration.

## INTRODUCTION

Many aposematic prey use visual warning signals, typically conspicuous coloration, to warn predators about their unprofitability, such as toxic or unpalatable chemical defenses (Poulton 1890). Predators need to learn to recognize warning signals, and conspicuous and consistent signals enhance this avoidance learning (Gittleman and Harvey 1980; Roper and Redston 1987). Selection by predators is therefore expected to lead to invariable warning signals, but many aposematic species show considerable intraspecific variation in their color patterns (Briolat et al. 2019). This is often explained by limits to optimal warning signal expression, including costs associated with signal production (Blount et al. 2012), and trade-offs with other functions of coloration, such as sexual selection (Maan and Cummings 2008) or thermoregulation (Lindstedt et al. 2009; Hegna et al. 2013). However, selection pressures from predators might also be more complex than traditionally assumed (Endler and Mappes 2004).

There is both within and between species variation in predator responses to aposematic prey (Endler and Mappes 2004), and spatial and temporal variation in predator pressure can lead to variable warning signals in prey (Nokelainen et al. 2014; Rönkä et al. 2020). Predator species may, for example, differ in their visual (Mochida 2011) and cognitive abilities (Rowland et al. 2017), or in their resistance to prey toxins (Fink et al. 1983; Brodie and Brodie 1990). In addition, individuals of the same predator species differ in their prior experience (Exnerová et al. 2007), personality (Exnerová et al. 2010), and current physiological condition (Barnett et al. 2007; Skelhorn and Rowe 2007), which can influence their decision to attack aposematic prey. This heterogeneity among predators can generate variation in selection pressure for signal conspicuousness and help maintain variable color patterns in aposematic species (Endler and Mappes 2004; Nokelainen et al. 2014; Rönkä et al. 2020).

Besides predator diversity, the avoidance learning process plays an important role in the evolution of warning signals (Skelhorn et al. 2016). Warning signals are often complex, and different signal components may elicit distinct predator responses, which might heighten selection on some elements, while relaxing selection on others (Winters et al. 2017). For example, many warning signals are multimodal, consisting of visual signals, sounds, odors, and chemical secretions that may have interactive effects (Rowe and Halpin 2013). Another potential factor influencing warning signal efficacy is prey shape or posture that might be particularly important when visual signals are combined with deimatic behavior (Hernández-Palma et al. 2023; Riley et al. 2023). Even within one modality, there can be multicomponent signals that consist of different elements, such as visual warning signals that are composed of distinct colors, shapes, and patterns on different body regions (Rowe 1999; Bradbury and Vehrencamp 2011). Predators might use several components when making foraging decisions (Pegram et al. 2013; Kikuchi et al. 2016), or alternatively base prey avoidance on a specific component, which could allow variation to exist in other color patterns (Winters et al. 2017). For instance, fish predators associate the yellow rim of nudibranchs with their unpalatability but do not learn to avoid nudibranchs based on their red spots, which might lead to relaxed selection on consistency of red-spotted patterns (Winters et al. 2017). Indeed, red spots were found to vary within and between nudibranch populations, whereas the yellow rim remained invariable (Winters et al. 2017). Considering the impact of individual color pattern elements on predator behavior may therefore help us to understand the maintenance of unexpected warning signal variation in aposematic species.

**Figure 1:**
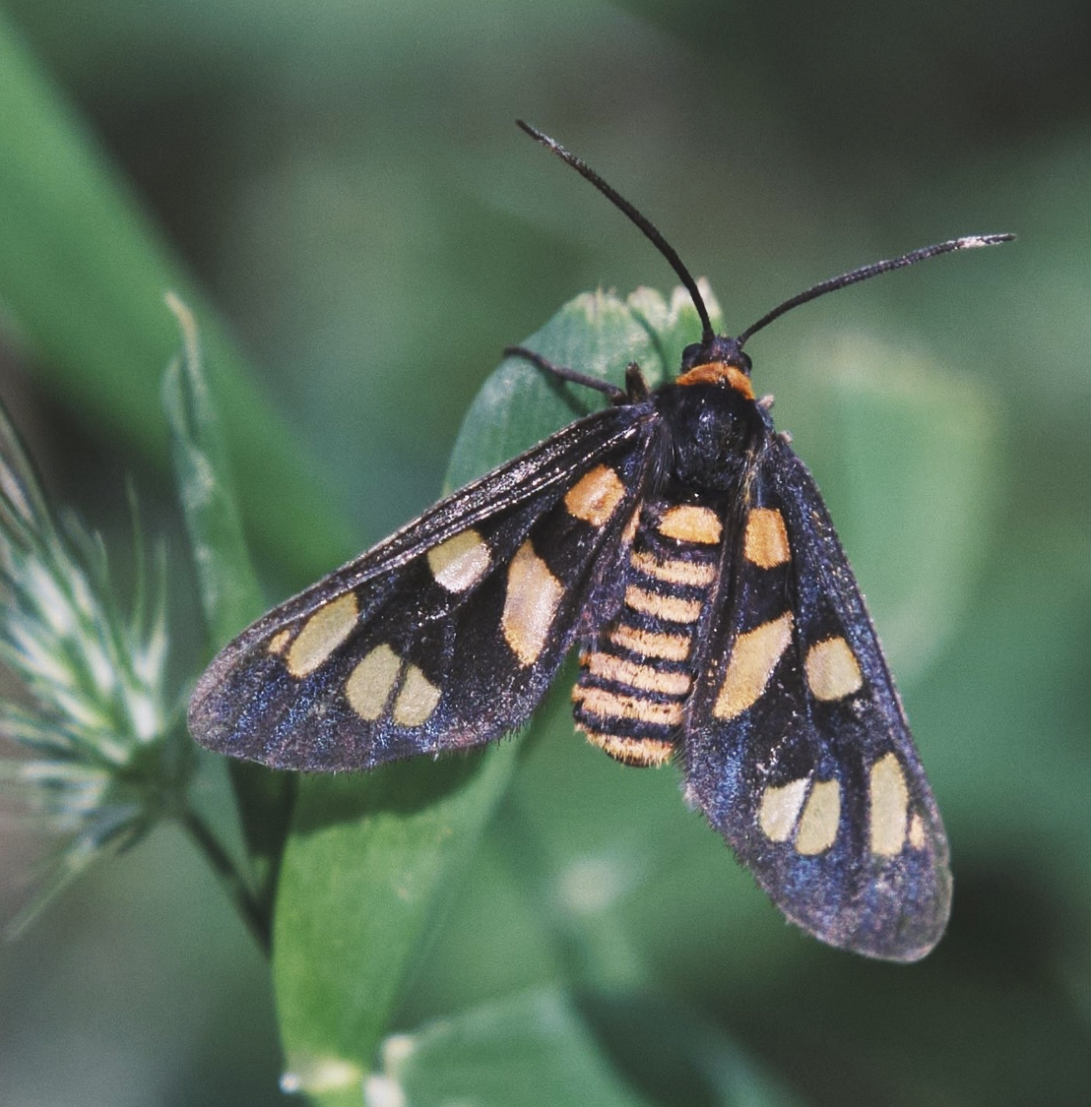
*Amata nigriceps* warning coloration, consisting of orange wing spots and orange stripes on the abdomen. Image: Yorick Lambreghts.

Here, we investigated how predators respond to color pattern elements of aposematic moths, *Amata nigriceps*, that are found along the east coast of Australia. The moths are chemically defended (Rothschild et al. 1984) and have black and orange color patterns on their wings and body. The wing patterns include orange spots against a black background, and the body coloration consists of orange and black stripes on the abdomen (Figure 1). Both wing spots and abdominal stripes could function as a warning signal, but their relative importance on predator attack decisions has not yet been tested. The two color patterns also differ in visibility: when the moths are resting, the wings can cover a large proportion of the abdomen, making the stripes invisible. The size of the orange spots on the wings is highly heritable and varies from 10 to 30% of the wing area within and across populations, and females typically have larger orange spots than males (Binns et al. 2022). Orange stripes on the abdomen, in contrast, are more consistent, with each sex having a fixed number of stripes in abdominal segments (females: five orange stripes; males: six orange stripes) and low variation in stripe width (Binns et al. in preparation). What role predation plays in the maintenance of this variation in the wings and consistency in abdominal stripes among individuals, however, remains untested.

We conducted predation trials in the field to test how orange wing spots and abdominal stripes influence predator foraging decisions. We used artificial moth models and noisy miners (*Manorina melanocephala*) as predators. Predation attempts on *A. nigriceps* are difficult to observe in the wild and their main predators are therefore unknown, but noisy miners are generalist feeders that regularly incorporate moths into their diet (Higgins et al. 2006). The experiments were conducted during the *A. nigriceps* flight season in locations where both *A. nigriceps* and noisy miners are commonly found, so birds were likely to have encountered the moths before the trials. We conducted three different experiments where birds were presented with two-choice preference tests. In Experiment 1, our aim was to investigate whether birds discriminate between moths with small and large wing spots when the effect of abdominal stripes was removed by painting the model abdomens black. In Experiment 2, we tested biases toward the same small versus large wing spots but this time the model abdomens included orange stripes to investigate if this changed how birds perceived the wing signals. In Experiment 3, both moth models had the same size wing spots, but we manipulated the number of abdominal stripes to investigate their effect on predator foraging decisions.

## METHODS

### Predator species and locations

We conducted field experiments with noisy miners between September 2020 and March 2022 with the permission from the Animal Ethics Committees at Macquarie University (ARA 2020/009) and at the University of New England (AEC20-099). Experiment 1 was conducted in two field sites: Macquarie University campus, Sydney, NSW (Wallumattagal Land, 33°46ʹ26″ S 151°06ʹ46″ E), and Newholme Research Station of the University of New England, Armidale, NSW (Anēwan Land, 30°25ʹ26″ S 151°39ʹ13″ E). Experiments 2 and 3 were conducted only on Macquarie University campus. The noisy miner is a medium-sized honeyeater endemic to southeastern Australia (Higgins et al. 2006). The species is a generalist forager that feeds on nectar, fruits and insects. The breeding season occurs from June to December and can include several broods (Higgins et al. 2006). Noisy miners are inquisitive, and readily explore new objects and become habituated to humans quickly in urban areas. They live in large colonies that have complex social structures, and individuals in the colony are usually found in the same geographical areas (Dow 1979). The mean diameter of these “activity spaces” averages 114 m for males and 74 m for females (Dow 1979). To minimize the likelihood of testing the same individuals several times, we chose test locations that were at least 500 m apart on Macquarie University campus. The test locations at Newholme Research Station were closer to each other (approximately 250 m), but there the majority of the birds were color banded and we could individually identify birds to ensure that the same individual did not visit multiple locations.

### Artificial moths

#### Wings

**Figure 2:**
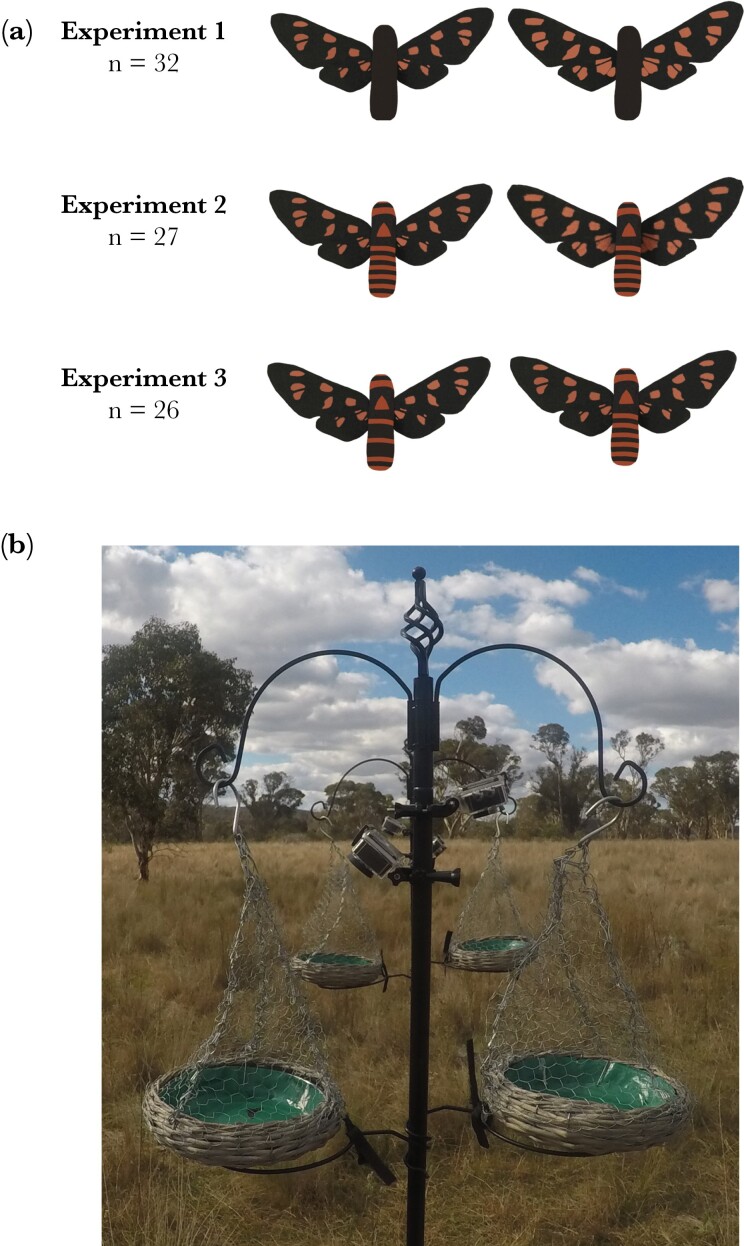
Experimental set-up for predator trials. (a) We conducted three separate experiments where birds were presented with a pair of moth models. In Experiment 1, the models had small versus large wing spots but no abdominal stripes, in Experiment 2 models also had small versus large wing spots and we added the natural pattern of abdominal stripes and in Experiment 3 we kept the wing spot size constant but varied the number of abdominal stripes. (b) The moth models were presented on four feeding trays that were surrounded by wire mesh and attached to two poles. Birds could enter only from one side of the feeder and their choices were recorded with small cameras attached to the poles.

For all three experiments we used the same methods to make the wings. We created artificial moths with “small” and “large” orange wing spots that represented the observed variation in warning signal size among 200 *A. nigriceps* collected from Sydney, Australia, between years 2018 and 2019 (Binns et al., in preparation). We designed signals to match the lower and upper quartiles of this variation, resulting in 15.5% of the wing area orange in the small and 22.1% of the wing area orange in the large spot (Figure 2a). Similar variation in wing signals is observed in the moth population on Macquarie University campus (Binns et al. 2022) and we expect to find both signal types also in our other study location at Newholme Research Station, although this has not been quantified. The model wings were created from images of real *A. nigriceps* moths that represented small (15.5.% orange) and large (22.1% orange) wing spots, using Adobe After Effects CS4 (Christiansen 2013). The total number of spots was held constant at 22. We used average wing length (15 mm; Binns et al., in preparation), and the same wing shape for both signals (see Supplementary Material for details of model preparation). The finished wings were duplicated and horizontally flipped to obtain symmetric left and right wings. These were printed on Kodak matte photo paper, using an Epson Stylus Photo RE3000 printer and Genuine Epson 157 ink. To ensure that orange wing spots matched the real color of *A. nigriceps* moths as closely as possible, we chose the orange color based on the color reflectance values of orange wing spots of real *A. nigriceps* moths (Supplementary Figure S1a).

#### Bodies

We made the model abdomens differently to suit the questions for each of our three experiments. In Experiment 1, abdomens were left plain black to isolate the effect of the wing spots. Our pilot studies suggested that birds would not attack moth models with clay bodies. We therefore increased birds’ motivation to attack models by making moth bodies of cake (Woolworths Madeira Cake) that has been used successfully as a palatable reward in previous studies with noisy miners (Farrow et al. 2017), and by pre-training the birds to visit feeders containing cake (see below). To make the bodies black, we mixed 10 g of cake with 2 mL of black food dye (Queen Classic Black Food Color). We then used this mixture to prepare 12 mm long and 3 mm wide bodies (weight 0.3 g ± 0.05 g) that resembled the real size of *A. nigricep*s moths (Binns et al., in preparation). These were placed between the paper wings.

In Experiments 2 and 3, we tested how birds responded to moth models when body coloration resembled the real coloration of *A. nigriceps* (Figure 1). This included adding an orange triangle to the thorax, five orange stripes to the abdomen and one orange stripe behind the head, as well as orange tip to the abdomen (Figure 2a). For these two experiments, the bodies were made using Monster Clay® medium modeling clay (The Monster Makers, Ohio, USA) as it was not possible to paint orange markings on cake. The clay was heated up to a liquid, poured into 12 mm long and 3 mm wide molds and allowed to set for 24 h (Binns et al., in preparation). The bodies were then painted black and orange using MontMarte® Acrylic paints, using a mixture of paints closest to the orange stripes of the real moths, based on the color reflectance measurements (see Supplementary Figure S1b). In Experiment 2, all model bodies included five stripes on the abdomen. For Experiment 3, we painted half of the bodies with only three abdominal stripes and half of the bodies with five abdominal stripes (Figure 2a) to test whether birds use the number of stripes as a cue in their foraging decisions. The width of the stripes was the same in bodies with three and five stripes and was based on the measurements from real moths (0.5 mm, Binns et al., in preparation). The painted bodies were glued to the paper wings using a non-toxic UHU glue stick. Because we found that birds did not attack these clay bodies, a piece of cake (similar shape and size used in the Experiment 1 but not colored black) was placed under the moth models to encourage birds to choose between the two options. Even though there was a slight change in the experimental design between Experiment 1 and Experiments 2 and 3 (cake being between the wings or under the clay body), we found that birds paid attention to the signal elements in both cases (see Results), and this methodological difference was therefore unlikely to influence our results.

### Pre-training and experimental setup

Before the experimental predation trials, birds were trained to visit round feeding trays (17cm diameter) that were surrounded by wire mesh with one opening where the birds could enter (Figure 2b). This ensured that only one bird at a time could visit the feeder, and that birds always approached the moths from the same direction. We used four feeding trays that were attached to two feeding poles (approximately 80 cm from each other; Figure 2b), so that several birds could be trained and tested simultaneously. We started to train the birds to visit the feeders approximately 10 days before the experiment by offering them pieces of cake until birds were habituated to the feeders and approached them immediately when the food was presented (approximately after 5 days of training). After the birds were trained to the feeders, they were further trained on stimuli relevant to Experiments 1, 2, or 3. Depending on the experiment for which the birds were being trained, birds learned either to eat cake pieces that were colored black with food dye (Experiment 1), or to find cake pieces placed under a piece of brown paper that was similar shape to the moths used in the experimental trials (Experiments 2 and 3). The birds were deemed “trained” when they readily consumed the black cake or had learned to find the cake from under the brown paper. This took approximately 5 days to achieve and at the end of the training birds typically flew to the feeders immediately after they were presented and ate the cake without hesitation.

### Predation trials

We conducted three different predation experiments with artificial *A. nigriceps* moths to investigate predator responses to Experiment 1) small versus large orange wing spots with black abdomen, Experiment 2) small versus large orange wing spots with striped abdomen, and Experiment 3) low versus high number of abdominal stripes with wing spots held constant (small spots, Figure 2a). In each experiment, we recorded the first choice of the birds (which cake piece was eaten first). The latency to attack the models was not analyzed because it was not possible to tell from the videos the exact time when birds saw the models, and both signal types were not necessarily attacked during the same visit to the feeder (both models attacked: *n* = 47, only one model attacked: *n* = 38).

#### Experiment 1: The effect of wing spots without abdominal stripes

The first experiment was conducted on Macquarie University campus from September 2020 to February 2021, and at Newholme Research Station in April 2021. At each field site, we conducted foraging trials in eight different locations. In the trials, a pair of moth models with small and large spots were presented to birds on four feeding trays (Figure 2b). The paper models were glued to a green background (green paper attached to the tray) 3.5 cm apart from each other, randomizing their side in each trial, and the black cake body was added between the wings.

The birds were observed from a distance (approximately 5 m), and their choices were recorded using small video cameras (Action Camera, Muson 4K) attached to the feeding poles (Figure 2b). We recorded the birds’ choice when they encountered the moths for the first time because subsequent contact could lead the bird to learn that the cake between the moth wings is palatable regardless of the signal size and change their response to the signals. As the birds on Macquarie University campus were not individually identifiable, we could not distinguish different individuals that were visiting the feeders. To minimize the likelihood of testing the same individuals twice, we only used the data from birds that arrived first in each test location (1–4 birds per location, depending on how easy it was to follow and distinguish them from each other). At Newholme Research Station, nine of the birds that visited the experimental setup were color-banded, so we could identify them from the video recordings. Birds that were not banded (*n* = 6) were included in the data only if they were the first 1–4 birds per location (following the same protocol as on Macquarie University campus). The feeders were recorded for 30 min on Macquarie University campus and for 60 min at Newholme Research Station, because at Newholme the color bands enabled us to identify individuals that arrived at the feeders later. The moth bodies were replaced each time birds visited the feeders, so that birds always had a choice between the two signals. We recorded the first choices of 32 birds that we could confidently identify as different individuals (Macquarie University campus: *n* = 17, Newholme Research Station: *n* = 15).

#### Experiment 2: The effect of wing spots and abdominal stripes

The second experiment was conducted in 10 different locations on Macquarie University campus from October to December 2021. Seven of these locations were the same as in Experiment 1, and it is therefore likely that some birds participated in both experiments, which could have influenced their responses. However, birds were exposed to the models for only 30 min, and there were 8 months between the two experiments, so the groups were likely to include new individuals and any “repeat birds” did not have recent experience of the models. We used the same wing patterns as in Experiment 1, but this time the moth bodies were made of clay and featured abdominal stripes (Figure 2a). We followed the same methods as in Experiment 1, but the moth models were glued to the background so that the clay body was lifted up slightly and a piece of cake could be placed under it. To qualify as a first choice, birds had to eat the cake under the model. We followed the protocol from Experiment 1 to record the first choices of birds that we could identify as different individuals, which resulted in a sample size of 27 birds.

#### Experiment 3: The effect of abdominal stripes

The third experiment was conducted on Macquarie University campus from February to March 2022. We used the same 10 test locations as in Experiment 2, so most birds were likely to have experienced the models before, with the addition of young from the 2021–2022 breeding season. For those birds that had experienced the models before, the most recent exposure to the models was at least three months prior to Experiment 3. We offered birds two moth models that had the same small orange wing spots, but that differed in the number of abdominal stripes, having either five or three stripes (Figure 2a). We followed the protocol from Experiment 2, placing a piece of cake under the moth models and recording which one was eaten first. As in previous experiments, we only used the first choices of the birds that we were confident to be different individuals (*n* = 26).

### Statistical analyses

We investigated whether birds had preferences toward the different signal types using generalized linear models with a binomial error distribution. The order in which the models were attacked (which cake was eaten first) was used as the response variable and this was explained by the signal type (Experiments 1 and 2: size of wing spot, small and large; Experiment 3: number of stripes, low and high); and the side of the tray (left and right). Because the trials in Experiment 1 were conducted at two sites (Macquarie University campus and Newholme Research Station), we also included an interaction between site and signal type to test for any differences in bird responses between the sites. Nonsignificant interaction was removed from the final model (see Results), but both main effects (signal type and side of tray) were retained in the models regardless of their significance. All analyses were conducted using R version 3.6.1 (R Core team 2019). The graphs were made using the package ggplot2 (Wickham 2016).

## RESULTS

### Experiment 1: The effect of wing spots without abdominal stripes

**Figure 3:**
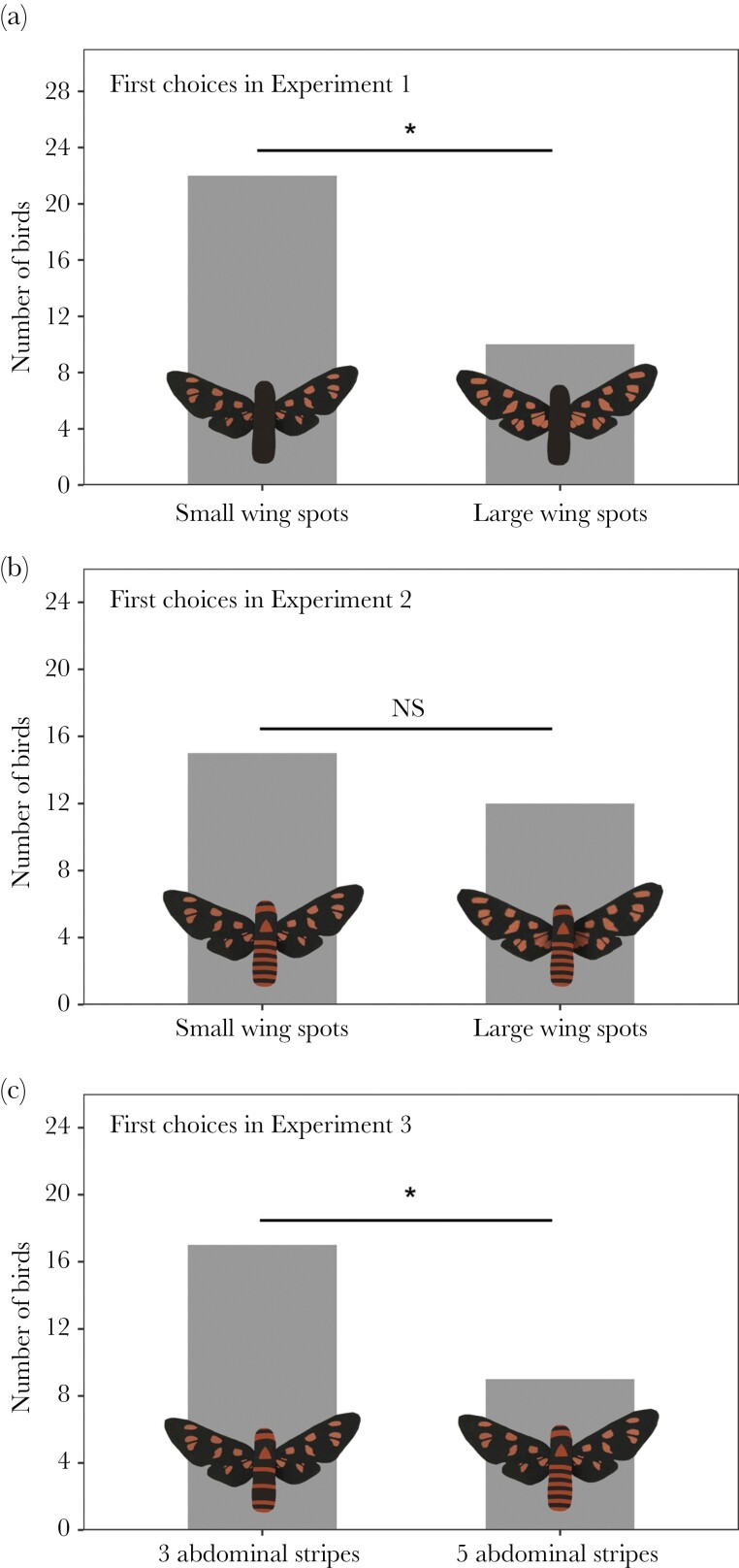
Birds’ first prey choices in the experiments. (a) In Experiment 1 (*n* = 32), moth abdomens were black and birds were more likely to attack a moth model with small wing spots. (b) In Experiment 2 (*n* = 27), wing spots were combined with abdominal stripes and there were no significant biases toward small versus large wing spots. (c) In Experiment 3 (*n* = 26), birds were more likely to attack a moth with three abdominal stripes compared to a moth with five stripes. (*P < 0.05).

The choices of the birds were similar in both test sites (signal choice × test site: estimate = 0.791 ± 1.096, Z = 0.721, *P* = 0.47), so we removed “test site” from the final model. We found that birds were more likely to attack a moth model with small wing spots as their first choice, compared to a moth with large wing spots (estimate = −1.609 ± 0.548, Z = −2.938, *P* = 0.003; Figure 3a). There were no biases toward prey on the left or right side of the tray (estimate = 0.223 ± 0.548, Z = 0.407, *P* = 0.68).

### Experiment 2: The effect of wing spots and abdominal stripes

When presented with moth models that included orange abdominal stripes, birds no longer had a significant preference for small wing spots (estimate = 0.406 ± 0.553, Z = 0.734, *P* = 0.46; Figure 3b). Similar to the first experiment, the side of the tray did not influence the initial attack choices (estimate = 0.406 ± 0.553, Z = 0.734, *P* = 0.46).

### Experiment 3: The effect of abdominal stripes

There was a significant effect of abdominal stripes on bird choices, with a higher number of birds attacking the moth model that had only three orange stripes on the abdomen (compared to moths with five stripes: estimate = −1.386 ± 0.607, Z = −2.285, *P* = 0.022; Figure 3c). Again, there were no biases toward prey on the left or right side of the tray (estimate = 0.575 ± 0.607, Z = 0.948, *P* = 0.34).

## DISCUSSION

Aposematic animals often have multicomponent warning signals, and understanding the selection pressures that maintain warning coloration requires exploring the relative importance of each component on predator foraging decisions (Winters et al. 2017). Here, we investigated how avian predators respond to two warning signal components of *A. nigriceps* moths: orange wing spots and orange stripes on the abdomen. Moths with small wing spots were more often attacked first by predators compared to those with large wing spots, but only when orange stripes on the abdomen were not visible. We also found that the number of orange stripes on the abdomen influenced predators’ foraging decisions, with birds being more likely to attack a moth model with a smaller number of stripes. Our results therefore suggest that orange stripes in *A. nigriceps* are an important warning signal component and a primary cue for predators whereas wing spots are used only when abdominal signals are not available. This could reduce selection on consistency in wing patterns, and as such our study provides further support for the idea that warning signal variation can be explained by differential selection pressures on individual color pattern elements (Winters et al. 2017).

Different components of aposematic signals vary in their importance in discrimination learning and generalization, and predators often base their foraging decisions on high-salience traits compared to less salient ones (Bain et al. 2007; Kazemi et al. 2014; Kikuchi and Sherratt 2015; Sherratt et al. 2015). For example, color is typically found to be a more important cue for predators than patterns or prey shape (Gamberale-Stille and Guilford 2003; Aronsson and Gamberale-Stille 2008; Kazemi et al. 2014; Sherratt et al. 2015; Riley et al. 2023; but see Lee et al. 2018; Linke et al. 2022). Similarly, different color pattern elements may differ in their salience, and predators can associate prey defense with one specific color pattern (Kikuchi et al. 2016; Winters et al. 2017). This seemed to be the case in our study where abdominal stripes overshadowed the effect of wing spots, suggesting that the stripes are a primary cue for predators. This result might have been influenced by our study design where birds consumed only the moth body (or a cake under it), which could have directed their attention to the abdomen coloration, but this is unlikely because bird did base their attack decisions on the wing signal in Experiment 1. Furthermore, because predation attempts on *A. nigriceps* are difficult to observe in the wild, we do not know where birds usually target their attacks, and which parts of the moths (if any) are consumed. Since we conducted our experiments in the field, the previous experience of the specific noisy miners involved is also unknown. However, the timing of the different experiments overlapped, so the number of less experienced juveniles was likely to be similar in each experiment. In addition, we conducted all the experiments during the *A. nigriceps* flight season and the moths commonly occur in the study areas, so we assume that most birds had encountered them previously.

Alternatively, it is possible that predators make their attack decisions based on the overall conspicuousness of the prey (Dreher et al. 2015), and specific color pattern elements are less important. This might provide another explanation for our results because adding orange abdominal stripes reduced the difference in conspicuousness between the two wing signals we tested, and could therefore explain why birds did not discriminate between them. It is also possible that after reaching some threshold value in the overall proportion of orange, slight variation in wing coloration no longer affects predator foraging decisions, perhaps because the prey is perceived to be too toxic to attack. In some species, more conspicuous warning signals are associated with higher toxicity (i.e., honest signaling, Summers et al. 2015), which could explain predator decisions to choose a less conspicuous alternative. However, there is no evidence of an association between wing coloration and toxicity in *A. nigriceps* (Hämäläinen et al. in preparation). Similarly, there is no evidence of toxicity differences between the sexes (Binns et al., in preparation), even though females have larger orange wing signals than males (Binns et al. 2022). Females and males also differ in the abdominal patterns, with males having one more orange stripe (six stripes) than females (five stripes), but this does not influence the proportion of orange on the abdomen that is similar in both sexes (Binns et al., in preparation). Because our aim was to test how the amount of orange in each color pattern influences predator responses, we manipulated both the number of stripes and the proportion of orange on the abdomen, and it is not possible to disentangle these two effects. Testing the effects of the pattern and overall conspicuousness separately therefore provides a prospective area for future research.

Although orange abdominal stripes seemed to be a primary cue to predators, we also found that the wing spots were important when the stripes were not visible. This leads to the question—how visible is each warning signal element when predators encounter the moths? First, signal visibility is likely to depend on prey behavior and posture. In general, moth hindwings are normally hidden during rest and the warning signals in hindwings are visible only when moths open their wings (Kang et al. 2017). However, *A. nigriceps* has orange wing spots on both their fore- and hindwings, so even if hindwings are hidden when the moths are resting, the warning signals in the forewings remain clearly visible (Figure 1). The visibility of the orange abdominal stripes during resting behavior is less straightforward: the stripes can be completely or partly covered by the forewings, or completely visible (L. Hämäläinen, personal observation, Figure 1), but how common each of these resting postures is remains unknown. Similarly, we know little about how predators perceive the different warning signal elements when the moths are flying. In some cases, color patterns appear to blur when prey move with sufficient speed (flicker fusion effect; Titcomb et al. 2014; Umeton et al. 2019). The abdominal stripes of *A. nigriceps* could create this effect during flight, and in this case the overall color ratio of black and orange might be more important than the striped patterns. However, the moths appear to have a slow flight pattern (L Hämäläinen, personal observation), although their escape flight speed and the visibility of abdomen during flight sequence has not been quantified. Future work should therefore aim to determine the visibility of each warning signal element during rest and flight as this is essential for understanding their importance in predator decision-making.

How predators perceive different warning signal elements also depends on the viewing context, such as distance to the prey and visual environment (Ruxton et al. 2018). Many color patterns are visible only when predators are in close proximity to the prey (Barnett et al. 2018a, 2018b). For example, orange and black stripes of aposematic cinnabar moth (*Tyria jacobaeae*) caterpillars are salient at close range, but the patterns blend into the background when viewed from a distance (Barnett et al. 2018a). Similarly, the different elements of *A. nigriceps*’ warning signal might be salient only when predators are very close to the moths, and these distance-dependent effects require further investigation. Another important factor that may influence warning signal detectability and predator responses is the light environment (Rojas et al. 2014). For example, birds choose to attack different color morphs of an aposematic wood tiger moth (*Arctia plantaginis*) depending on the light conditions (Nokelainen et al. 2022). Heterogeneity in the light environment and background might therefore influence the salience of the different warning signals elements, and their importance for predators could be context dependent. Finally, our experiment included only visual warning signals. However, in many aposematic species these are combined with other signal modalities, such as odors or chemical secretions (Rowe and Haplin 2013), which can have interactive effects that change predator responses to visual signals (Rojas et al. 2019). This might be the case also in *A. nigriceps* that secrete defensive neck fluids when attacked (Binns et al. 2022). These secretions could include odor cues, and further research is needed to understand the potential interactions between different signal modalities, and whether this changes predator responses to different visual elements of *A. nigriceps* warning signals.

Our study demonstrates that different warning signal elements may vary in their salience to predators, and understanding selection pressures for prey warning coloration requires investigating the function of each individual element (Winters et al. 2017). We show that the orange abdominal stripes of *A. nigriceps* are an important warning signal for noisy miners, which could lead to relaxed selection on orange wing spots and provide one explanation for the variation in the wing spot size (Binns et al. 2022). However, predators used the wing signal in their foraging decisions when the stripes were not visible, and future work should aim to quantify the visibility of each warning signal element in different contexts to understand their role in predator attack decisions. For example, it is possible that the orange wing spots have protective value when the moths are resting and their abdomen is not visible, whereas the abdominal stripes could be a more salient cue during flight. Both color patterns could also have other functions than warning coloration. Wing spots, for instance, could function as disruptive coloration, which might provide another explanation for variable wing patterns, and the role of wing spots and abdominal stripes of *A. nigriceps* in contexts other than antipredator defenses, such as in sexual selection, remains uninvestigated. Overall, our study suggests that examining the individual roles of warning signal components may change our predictions of the evolution of prey warning coloration and help understand the observed diversity of warning signals in nature.

## SUPPLEMENTARY MATERIAL

Supplementary material can be found at http://www.beheco.oxfordjournals.org/

arad097_suppl_Supplementary_Material

## Data Availability

Analyses reported in this article can be reproduced using the data provided by Hämäläinen et al. (2023).
