# Supplementary material for: Predator selection on multicomponent warning signals in an aposematic moth
Source: Behav Ecol. 2023 Nov 16;35(1):arad097. doi: 10.1093/beheco/arad097 (PMC10976905; doi:10.1093/beheco/arad097)
Supplement: arad097_suppl_Supplementary_Material [file arad097_suppl_supplementary_material.docx]

**Supplementary Material for**

**Predator selection on multicomponent warning signals in an aposematic moth**

**Liisa Hämäläinen*, Georgina E. Binns, Nathan S. Hart, Johanna Mappes, Paul G. McDonald, Louis O’Neill, Hannah M. Rowland, Kate D.L. Umbers & Marie E. Herberstein**

*** Correspondence:**

Liisa Hämäläinen

Department of Biological and Environmental Science, University of Jyväskylä, Finland

Email: liisa.l.hamalainen@jyu.fi

**Details of preparing the artificial moths**

We followed the methods from Binns et al. (in prep.) to prepare the moth models used in the experiment. The models were created based on 200 *Amata nigriceps* individuals that were collected from Sydney, Australia, between 2018 and 2019. The moths were imaged and the proportion of orange in the wings was quantified following the protocol from Binns et al. (2022). The wing signals used in the experiment represented the variation in wing coloration of the collected moths, matching the lower (15.5% orange) and upper (22.1% orange) quartiles of this variation. The images were also used to quantify morphometrics (average body length and width, wingspan, and width of orange and black abdomen stripes) using ImageJ software (v1.52a), and the average morphometric measures were used for the model preparation (see main text for details). Next, we chose randomly 33 of the collected moths and measured their orange wing spots and body stripes using Ocean Optics USB4000-Fl spectrometer and a PX-2 pulsed xenon light source, calibrated against a Spectralon (Labsphere, Congleton, UK) white standard. The spectrophotometry measures of two orange abdominal patterns (a triangle in the thorax and the third abdominal stripe) and three largest wing spots from each moth were then pooled and averaged, and these were used as a color reference for the model wings and bodies (Figure S1).

***Wings***

We first selected real images of *Amata nigricep*s that represented small (15.5% orange) and large (22.1% orange) wing spots. These were modified using Adobe After Effects CS4. First, we applied a Tint effect, rendering the image greyscale. After that, we applied a Levels effect with Input Black set to 40 and Input White to 71, which made the orange spots white and the rest of the wing black. Any remaining small white specks on the wings outside of the spots were made black by placing a black solid layer over the wing and using the mask tool to select the areas to be rendered black. To ensure that the wing shapes were identical for the wings with small and large spots, the small spots were individually cut out using the mask tool and placed over a copy of the wing with large spots on which all the spots had been removed. We then created out a palette of orange/red (see below) and an orange solid layer was placed over each wing and the transfer mode set to Multiply, which caused the white spots to be colored orange. The wings were then duplicated and horizontally flipped to give both left and right wings. These were printed on Kodak matte photo paper, using an Epson Stylus Photo RE3000 printer and Genuine Epson 157 ink.

The colors used for the printed wings were chosen by comparing the color reflectance values of orange wing spots of real *A. nigriceps* moths to the values of the printed wings spots (both measured using the same spectrometer, see above). To compare the chromatic difference between the moth and the color swatches, we applied the Vorobyev–Osorio discrimination model (Vorobyev & Osorio, 1998) for a generalist bird insectivore, blue tit (*Cyanistes caeruleus*; LWS, λmax 563 nm; MWS, λmax 503 nm; SWS, λmax 448 nm; UVS, λmax 371 nm; double dorsal, DD λmax 563 nm; a Weber fraction value of 0.05 for the most abundant cone type), using the R package pavo (Maia et al., 2013).  The swatch that was indistinguishable from the moth was used to provide the RGB values to recolor the image of a single moth in Adobe photoshop (R208 G134 B80). To choose the color palette used for the orange wing spots, we used the same visual sensitivity model and calculated the Just Noticeable Differences (JNDs) of the real wing spectrometer readings and the printed wing colors (Figure S1A). We picked the orange/red palette with the lowest mean color contrast, rather than the lowest mean color luminance, because this visually looked like a closer match to real moth coloration.

***Bodies***

In Experiments 2 and 3, we made moth bodies using Monster Clay® medium modeling clay (The Monster Makers, Ohio, USA). The orange markings in the bodies were painted using a mixture of MontMarte® acrylic paints that was closest to the orange stripes of the real moths, based on the color reflectance measurements (Figure S1b). These were calculated using the same protocol described above, where the JNDs of the real moth abdomen coloration and orange paint mixes were compared using the blue tit visual sensitivity model. The color mix with the lowest mean color contrast was used for the experiment.


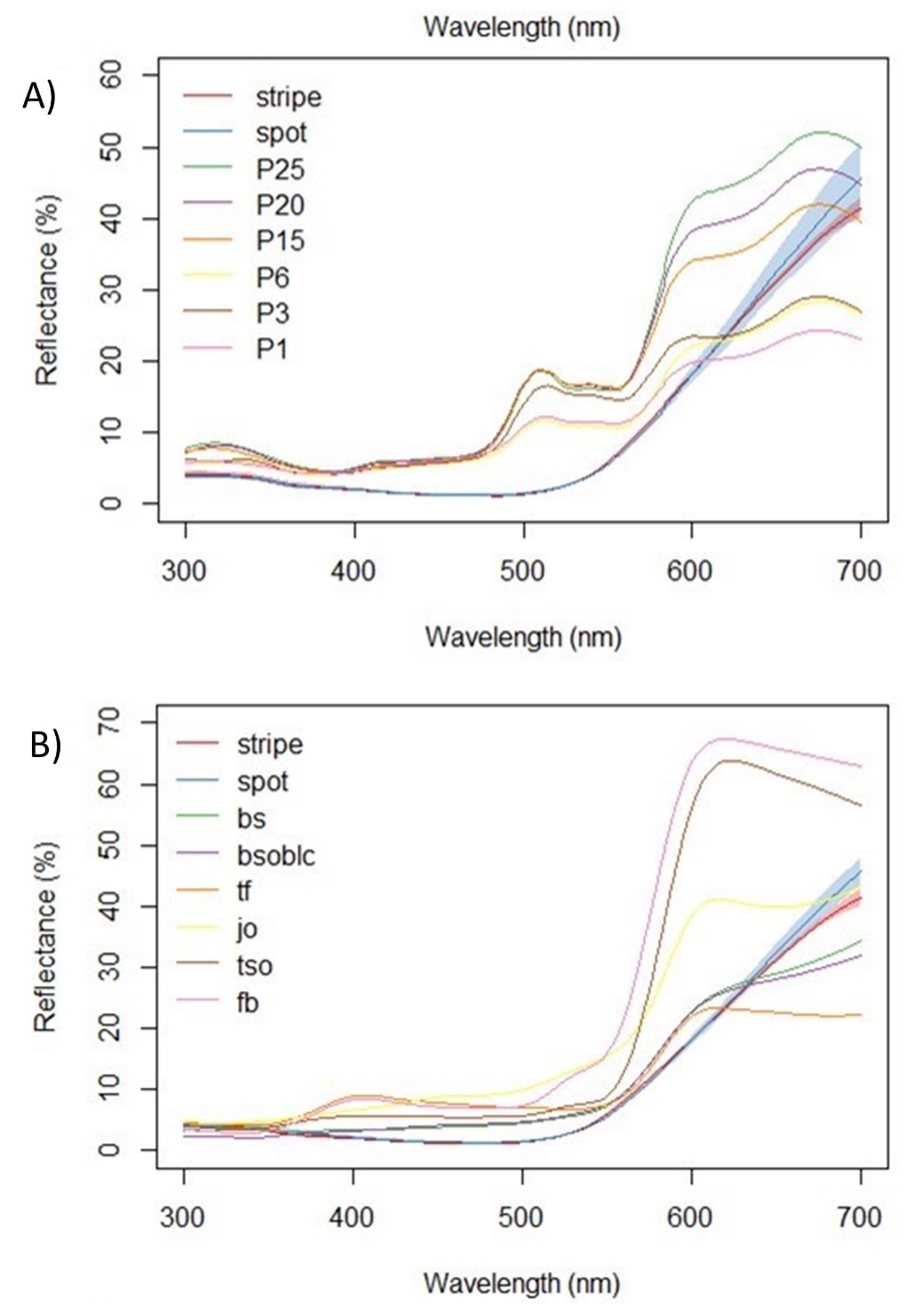


**Figure S1.** A) Spectrophotometric curves of *Amata nigriceps* averaged wing spots (‘spot’ - blue line, with 95% CI) and stripes (‘stripe’ - red line, with 95% CI), compared with the six closest matches of color palettes printed out on matte photo paper for the model wings (‘P1’ - ‘P25’). Color palette ‘P1’ had the lowest mean color contrasts compared to the averaged moth spots and stripes (2.7 JNDs) and we used it for printing the wings. B) Spectrophotometric curves of *Amata nigriceps* averaged wing spots (‘spot’ - blue line, with 95% CI) and stripes (‘stripe’ - red line, with 95% CI), compared with the six closest matches of model body paint mixes (‘bs’, ‘bsoblc’, ‘tf’, ‘jo’, ‘tso’, and ‘fb’). Spots and stripes had very similar color (0.37 JNDs) and luminance (0.08 JNDS). The paint mix ‘tso’ had the lowest mean color contrasts (3.9 JNDs) to the wing spot average and we used it for painting the orange markings in the clay bodies. Spectra graphs were made using R package ‘pavo’ (Maia et al., 2019).

**References**

Maia R, Gruson H, Endler JA, White TE. 2019. Pavo 2: New tools for the spectral and spatial analysis of colour in R. Methods Ecol Evol. 10:1097– 1107.

Vorobyev M, Osorio D. 1998. Receptor noise as a determinant of colour thresholds. Proc R Soc B. 265:351–358.
